# When effective anticancer therapies are, in fact, destabilizing the tumor’s Group Phenotypic Composition

**DOI:** 10.1038/s41698-026-01536-5

**Published:** 2026-06-03

**Authors:** Frédéric Thomas, Antoine M. Dujon, Andriy Marusyk, James DeGregori, Alexandre Fontanella, Margaux Bieuville, Mario Campone, Pascal Pujol, Catherine Alix-Panabières, Laurent Lecam, Benjamin Roche, Matthieu Lacroix, Christophe Hirtz, Laurent Poulain, Jean-Pascal Capp, Beata Ujvari, Jordan Meliani, Robert Noble, Aurora M. Nedelcu, Robert Gatenby

**Affiliations:** 1 CREEC/CANECEV, MIVEGEC (CREES) Department, University of Montpellier, CNRS, IRD, Montpellier, France; 2 School of Life and Environmental Sciences, Deakin University, Waurn Ponds, VIC Australia; 3 Department of Cancer Physiology, H Lee Moffitt Cancer Center and Research Institute, Tampa, FL USA; 4 Department of Biochemistry and Molecular Genetics, University of Colorado Anschutz Medical Campus, Aurora, CO USA; 5 Institute of Organismic and Molecular Evolution (iomE), Johannes Gutenberg-Universität, Mainz, Germany; 6 Institute for Quantitative and Computational Biosciences (IQCB), Johannes Gutenberg-Universität, Mainz, Germany; 7 Institut de Cancérologie de l’Ouest-René Gauducheau, Centre de Recherche en Cancérologie, Saint Herblain, France; 8 Oncogenetic Department, University Hospital of Montpellier, Montpellier; European Liquid Biopsy Society (ELBS), Hamburg, Germany; 9 Laboratory of Rare Human Circulating Cells and Liquid Biopsy (LCCRH), University of Montpellier, Medical Centre of Montpellier, Montpellier, France; 10 IRCM, Institut de Recherche en Cancérologie de Montpellier, INSERM U1194, Univ Montpellier, Institut régional du Cancer de Montpellier, Montpellier, France; 11 Equipe labélisée Ligue Contre le Cancer, Paris, France; 12 IRMB-PPC, INM, Univ Montpellier, CHU Montpellier, INSERM CNRS, Montpellier, France; 13 Inserm U1086 Anticipe et Plateforme ORGAPRED, Université de Caen Normandie,Centre de Lutte Contre le Cancer François Baclesse, Caen, France; 14 UNICANCER, Comprehensive Cancer Center François Baclesse, Caen, France; 15 Université de Caen Normandie, INSERM U1086 ANTICIPE (Interdisciplinary Research Unit for Cancers Prevention and Treatment) and PLATON Services Unit, ORGAPRED Core Facility, Caen, France; 16 Toulouse Biotechnology Institute, University of Toulouse, INSA, CNRS, INRAE, Toulouse, France; 17 Department of Mathematics, City St George’s, University of London, London, UK; 18 Department of Biology, University of New Brunswick, Fredericton, NB Canada

**Keywords:** Cancer, Computational biology and bioinformatics

## Abstract

Many cancer therapies achieve durable control without complete tumor eradication, suggesting that disrupting tumor organization may be more critical than killing cells. We propose that effective treatments converge by destabilizing the tumor’s Group Phenotypic Composition (GPC), the functional and spatial organization of interacting cell populations. When this organization collapses, tumors lose coherence. This perspective provides a unifying framework for designing therapies targeting tumor-level dynamics rather than cell number alone.

## Introduction

### From cell killing to system destabilization

Modern oncology remains dominated by the *maximum tolerated dose* paradigm^1,2^, grounded in the assumption that reducing the total number of cancer cells correlates directly with disease control. Yet, growing empirical evidence reveals paradoxical outcomes: some tumors relapse after aggressive therapy despite having regressed below the detection threshold, whereas others remain macroscopically detectable yet enter long-term dormancy or even regress following modest or intermittent interventions^3,4^. Indolent lymphomas and certain metastatic prostate or hormone-dependent breast cancers, for example, can remain clinically stable for years under limited^5^ or intermittent treatment (e.g., refs. ^6–8^), illustrating that long-term control does not necessarily require continuous cytotoxic pressure. These patterns suggest that the relationship between cytotoxic dose and durable control is not linear, and that therapeutic success cannot be fully explained by cell lethality alone. Indeed, over the past two decades, this logic has been questioned by evolutionary and ecological frameworks that emphasize competition, cooperation, and adaptation within the tumor ecosystem^9–11^.

We propose these paradoxes emerge from eco-evolutionary interactions among cancer cells and the way that therapies affect such interactions. This population-based eco-evolutionary framework suggests that the collective organization of tumors, the way heterogeneous cells interact, cooperate, and compete, plays a decisive role in progression and therapy response^11–17^. In this view, a tumor is not a mere aggregate of cells but a dynamic self-organized multicellular system evolving under *selection for function* at the group level^18^. We previously argued that the persistence of tumors depends on maintaining an oncogenic Group Phenotypic Composition (thereafter GPC, see also next section)^19^, corresponding to a dynamic configuration of interacting cellular phenotypes, spatial arrangements, and resource exchange systems that collectively determine tumor persistence/fitness under changing microenvironmental (including therapeutic) challenges (see refs. ^19,20^). Therapies that achieve disease control without eradicating all cells may act by disrupting this collective cooperative tumor architecture, pushing the system beyond a functional threshold and/or a size threshold (i.e., a tipping-point^21^), a phenomenon analogous to ecological collapse or phase transitions in complex systems (e.g., ref. ^22^). This GPC-based view extends existing ecological and evolutionary models by focusing on the stability of tumor-level organization rather than population composition alone, thereby linking phenotypic plasticity, cooperation, and resilience under therapy. Recognizing effective treatments as GPC-destabilizing interventions reframes therapeutic success not as maximal cell killing but as targeted disruption of tumor system stability, with broad implications for the rational design of dosing and combination strategies. For instance, adaptive therapy in prostate^23^ and breast^24^ cancer, intermittent targeted therapies in melanoma^25^, or immune checkpoint blockade^26,27^ can achieve durable control by perturbing intercellular dependencies rather than through direct cytotoxicity, consistent with GPC destabilization. A central question, however, is not whether therapies perturb tumor organization, but what distinguishes perturbations that lead to durable collapse from those that allow reorganization and relapse, a distinction that remains largely unresolved across current therapeutic paradigms. This question constitutes the central focus of this Perspective.

This paper proposes that many of oncology’s notable clinical successes, especially those that cannot be explained by cytotoxicity alone, are in fact instances of GPC destabilization. To our knowledge, no cancer therapy has been explicitly designed with the stated goal of destabilizing a tumor’s Group Phenotypic Composition (GPC) as a formally defined, system-level property. However, several therapeutic strategies have been intentionally developed to perturb specific components of tumor organization, such as cell–cell interactions, ecological competition, or spatial resource distribution. The key distinction is that these approaches were conceived to target particular mechanisms or interactions, rather than the stability of the tumor as an integrated, emergent system. In this sense, they were not designed to destabilize the GPC per se, but can be retrospectively understood as doing so. The GPC framework therefore does not reinterpret their intent but rather provides a unifying conceptual lens that connects these mechanistically distinct strategies through their shared effect on tumor-level organization and stability and argues for the continuing development of such therapies. Approaches such as adaptive therapy, differentiation therapy, immune checkpoint blockade, or vascular normalization, as well as more recent extinction or multi-strike strategies were explicitly conceived to disrupt competitive balances, cooperative interactions, or spatial and resource architectures within tumors, often by modifying cellular phenotypes or their interactions.

Our point is not to deny the existence of such strategies, but to emphasize that they have not been conceptualized within a unifying framework explicitly centered on tumor organization as an emergent property. The GPC framework provides such a perspective. It does not claim novelty in the existence of these approaches but offers a common conceptual language to understand why they work, how they might be deliberately optimized, and how similar principles could guide the design of new therapies. Furthermore, it highlights constraints and failure modes that are not readily apparent when these strategies are considered independently.

## Conceptual framework: the Group Phenotypic Composition and tumor functionality

### Defining the GPC

The Group Phenotypic Composition (GPC) framework, introduced in previous work^19^, and conceptually related to ecological frameworks describing group-level phenotypes and interaction networks (e.g., ref. ^28^), describes tumors as organized collectives defined by three interdependent dimensions: their cellular composition, spatial architecture, and functional interaction networks. In this Perspective, we build on this framework to examine how therapies reshape, destabilize, or reconfigure these organizational states. Together, these dimensions define the *functional phenotype of the tumor as a group*. In this framework, tumor functionality refers to the capacity of the system to sustain key collective processes, growth, invasion, immune evasion, and metabolic homeostasis, that ensure persistence in the host environment. These functions emerge from, and depend on, the integrity of the GPC.

Importantly, the GPC is not static but dynamically reshaped by environmental pressures, therapy, and internal regulatory dynamics, such as shifts in signaling networks, metabolic coupling, or cooperation and competition balances among cellular subpopulations. Through continual adaptation, tumors maintain metastable configurations that allow persistence under fluctuating conditions. In evolutionary terms, the tumor behaves as an evolving consortium under selection for function (sensu^29^): its success depends less on the fittest clone and more on maintaining a functional configuration that maximizes group-level persistence^18^.

Like other ecological communities, tumors maintain functionality through a balance of cooperation and competition; therapies that disturb this balance can trigger systemic collapse even in the absence of extensive cell death. If tumor organization is disrupted, e.g., by perturbing spatial order, altering proportions, or breaking key interactions, the collective can lose the emergent properties necessary for survival and/or progression, even though many constituent cells may remain viable. Although the GPC is a theoretical construct, its dimensions can increasingly be inferred from high-dimensional datasets generated by spatial transcriptomics, proteomics or metabolomics approaches as well as multiplexed imaging, and microenvironmental modeling^30^, which together offer experimental proxies for system-level organization and function.

### Critical transitions and tipping points

Complex biological systems often display non-linear responses: gradual parameter changes can lead to abrupt shifts in collective behavior^22,31^. Tumors, as eco-evolutionary systems, are likely governed by similar principles. Their GPC may remain stable within a robust configuration space, but once perturbations exceed a threshold, by modifying composition, spatial order, or environmental feedbacks, Thomas et al.^21^ have argued that the system may transition irreversibly toward dysfunction or extinction. Such parameters may include gradual shifts in resource availability, immune pressure, stromal activation, or drug-induced phenotypic turnover. In GPC terms, a critical transition reflects a loss of coordination among the compositional, spatial, or functional dimensions that normally stabilize the tumor’s collective phenotype.

These transitions often emerge from self-reinforcing mechanisms that amplify small imbalances, such as hypoxia-driven angiogenic loops, metabolic coupling between clones, or immune–tumor cell interactions, until the system reorganizes into a new stable configuration. Such dynamics correspond to well-characterized non-linear feedback processes, including positive feedback loops, bistability, and hysteresis, in which small perturbations can be amplified and lead to alternative stable system states. Whether the transition results in collapse or resurgence depends on the sign and context of the perturbation: when cooperative links are broken, the system may lose functionality, whereas their re-establishment or reprogramming can trigger renewed growth. In the context of the GPC framework, these alternative stable states can be interpreted as distinct organizational configurations of the tumor collective, differing in their capacity to maintain functional coherence.

Yet, critical transitions can also lead to the opposite outcome, namely a sudden *reawakening* or *reorganization* of the malignant collective^32^. Dormant or residual cell populations may switch to a proliferative state once ecological constraints are relaxed, as demonstrated in experimental studies of microenvironment-dependent metastatic dormancy and reactivation^4,33,34^. Similarly, paradoxical rebounds and increased invasiveness following anti-angiogenic therapies have been reported in experimental studies^35,36^, highlighting that the same principles of non-linearity can drive both collapse and resurgence^37^. Importantly, these non-linear dynamics are not limited to dormancy: macroscopic tumors may remain trapped in growth bottlenecks despite active proliferation due to spatial or stromal constraints, and later escape following discrete shifts in tumor–microenvironment interactions^38^.

Mathematical and empirical studies support this view. Models coupling tumor and immune dynamics exhibit alternative stable states and bistability^39–41^, which can be interpreted as transitions between distinct organizational configurations of the tumor collective within the GPC framework, and early-warning signals such as increasing variance or autocorrelation have been detected prior to tumor collapse^42–45^. These signals are characteristic of critical slowing down, reflecting a loss of system resilience as the GPC becomes increasingly sensitive to perturbations affecting its compositional, spatial, and functional organization. These features indicate that tumor collectives, like ecosystems (see for instance^46^), can cross tipping points where small therapeutic perturbations yield disproportionate outcomes. Understanding these critical thresholds offers a conceptual basis for therapies aimed not at maximal killing but at steering the tumor system toward irreversible GPC destabilization.

Importantly, tumor–immune interactions can themselves define such thresholds: therapies may induce regression not simply by cytotoxicity but by disrupting tumor-mediated immune suppression and breaking tolerance^47^. However, persistence can still occur if subsets of tumor cells re-establish immune tolerance, either through dormancy-like states or by evolving immune-evasion strategies such as loss or downregulation of antigen presentation machinery (e.g., refs. ^48–50^). These dynamics further illustrate that immune control and escape represent shifts between alternative GPC states, reinforcing the relevance of a threshold-based framework.

### Therapy as GPC modulation

From this perspective, any intervention, e.g., cytotoxic, metabolic, epigenetic, immune or ecological, can be reframed as an attempt to reshape the tumor’s GPC landscape. In practice, such reshaping occurs through altered selection pressures, disruption of metabolic and signaling dependencies, or reorganization of the spatial and immune architecture that supports group-level function. Traditional high-dose chemotherapy acts as a massive disturbance that may destroy the system entirely or select for a new, resistant GPC. Whether therapy acts as a destructive or modulatory agent depends on its intensity, timing, and the adaptive capacity of the tumor collective, which together determine whether the GPC reorganizes or collapses.

In contrast, therapies that modulate the GPC without complete destruction may guide the system toward a non-viable or unstable configuration, akin to an ecological regime shift. Crucially, the effectiveness of such therapies may not linearly correlate with tumor mass reduction but rather with the loss of internal functional coherence, including disruption of spatial patterning, resource networks, or cooperative modules. Yet, although not focusing on reducing tumor mass per se, GPC-based therapies can in some cases result in a significant decrease in tumor cell populations below what is referred to as the “Allee threshold”, leading to a spiral to extinction^51^. Importantly, GPC-destabilizing therapies do not necessarily rely on driving tumor populations below an Allee threshold. While such destabilization may in some cases induce population-level Allee effects and lead to extinction, it can also result in durable tumor control without eradication, by preventing the re-establishment of a functionally coherent collective capable of sustained progression. This outcome reflects fitness benefits (termed “Allee effects”) known to arise from interactions among individual members of a population and expected to increase as population size increases^52^. By increasing the global fitness of individuals within a population, Allee effects can promote population growth with range expansion (i.e., invasion^53^) and increase robustness to transient environmental and demographic perturbations^54^. Allee effects have been observed in cancers^38,55–57^, and may include neo-angiogenesis, reduction of predator-like effects of the immune system, and environmental changes (including acidification) promoting invasion and reducing immune recognition^58^, suggesting that cancer populations are susceptible to “Allee thresholds”. In fact, this idea underlies a recently proposed therapeutic strategy known as extinction therapy (ref. ^23^; discussed later). Importantly, Allee effects are conceptually distinct from GPC destabilization: they operate at the population level through density-dependent fitness, whereas GPC destabilization reflects the breakdown of collective functional organization. However, the two processes may interact when therapies simultaneously reduce population size and disrupt group-level functionality.

Overall, the GPC framework reframes several established strategies, including immune-mediated therapies, adaptive therapy, differentiation therapy, vascular normalization, and extinction or multi-strike strategies, as different ways of destabilizing the GPC. Recognizing these therapies as GPC modulations suggests that treatment design should aim to steer collective dynamics rather than merely eliminate cells, emphasizing controllable transitions and measurable indicators of system destabilization. This perspective opens a path toward predictive frameworks where therapy outcomes are evaluated through dynamic markers of GPC stability and resilience, rather than static measures of tumor size.

A key implication of the GPC framework, which has not been explicitly articulated in previous work, is that not all perturbations are equivalent in their consequences. This distinction provides a first step toward an operational framework for predicting therapeutic outcomes. While virtually any intervention can alter the tumor’s GPC to some extent, only a subset of perturbations lead to durable control. A key challenge is therefore to distinguish between superficial, reversible disruptions and those that irreversibly compromise the tumor’s functional organization. From this perspective, we propose that successful GPC-destabilizing therapies share several key features. First, they tend to target critical functional interactions within the tumor system, such as cooperative metabolic exchanges, immune suppression circuits, or spatially structured resource gradients, rather than merely reducing cell numbers. Second, they push the system beyond thresholds where reorganization becomes difficult or impossible, thereby preventing the re-establishment of a functionally coherent collective. Third, their effects are often temporally structured in a way that exploits transient vulnerabilities, for example by preventing the recovery of cooperative networks between successive perturbations.

In contrast, many therapies may perturb the GPC without producing durable effects because they fail to disrupt these key interactions, remain below critical thresholds, or allow sufficient time for the tumor to reorganize into a new stable configuration. In this sense, therapeutic failure can be understood as incomplete GPC destabilization, where perturbations alter tumor organization without irreversibly disrupting its core functional dependencies. This distinction may help explain why certain interventions, such as anti-angiogenic therapies or stromal depletion strategies, can produce transient responses followed by rebound or even accelerated progression. This framework shifts the focus from whether a therapy affects the GPC to how it does so, and highlights the importance of identifying the specific structural and functional vulnerabilities that govern the stability of tumor organization. Together, these observations suggest that the clinical outcome of GPC perturbation depends less on its occurrence than on its ability to irreversibly disrupt key functional dependencies within the tumor system.

From a clinical perspective, this framework indicates that effective therapy design may not require maximal cytotoxicity, but rather the minimal perturbation necessary to destabilize the tumor’s collective architecture. This shift could help reduce dose-limiting toxicities by prioritizing strategies that disrupt key cooperative interactions, spatial structures, or resource flows within the tumor. In this view, treatment optimization would focus on identifying combinations and dosing schedules that push the tumor across critical GPC thresholds, rather than maximizing tumor shrinkage alone. Importantly, this perspective also opens the possibility of developing biomarkers based on system-level instability, enabling clinicians to monitor when a tumor is approaching a tipping point and adjust therapy accordingly.

## Five therapeutic paradigms that destabilize the GPC

In this section, we revisit five therapeutic frameworks through the lens of GPC destabilization (See Table 1), proposing that each works because it represents a distinct pathway through which the tumor’s internal organization can be pushed beyond a critical threshold, even though it was not originally designed to achieve tumor destabilization.

**Table 1:** Therapeutic paradigms and their underlying mechanisms seen through the lens of GPC destabilization

Therapeutic paradigm	Primary GPC axis targeted	Mechanistic principle	Type of destabilization	Example
Immune-mediated collapse	Interactional & Network	- Transforms tumor interactions from cooperative to antagonistic - Promotes immune infiltration and feedback inversion	Interactional destabilization	Immune checkpoint blockade (e.g., anti-PD-1/PD-L1)
Adaptive therapy	Compositional	- Maintains competitive balance between sensitive and resistant clones - Prevents dominance of resistant lineages	Compositional destabilization	Intermittent low-dose chemotherapy in prostate cancer
Differentiation/phenotypic reversion therapy	Phenotypic & Functional	- Induces differentiation or partial normalization - Dissolves hierarchical and cooperative modules	Phenotypic/Functional destabilization	Retinoic acid therapy in acute leukemia
Vascular/microenvironmental normalization	Spatial & Ecological	- Reconfigures spatial and resource architecture - Removes ecological niches supporting resistance	Spatial destabilization	Anti-VEGF therapy in solid tumors
Extinction/multi-strike strategies	Compositional & Functional	- Sequential perturbations prevent reassembly of tumor organization - Pushes system below viability thresholds	System-level collapse	Sequential or adaptive multi-strike regimens

This table summarizes five major therapeutic frameworks reinterpreted in the framework of Group Phenotypic Composition (GPC) destabilization. Each paradigm targets a distinct organizational axis of the tumor, i.e., compositional, phenotypic, spatial, or interactional, yet all converge toward the same emergent outcome: the collapse of collective functionality. Mechanistic principles underlying each paradigm are listed to illustrate how specific interventions disrupt the corresponding GPC axis. Viewing these established therapies under a unified GPC framework emphasizes that durable tumor control often results not from total cell eradication, but from the loss of systemic coherence within the malignant consortium. Illustrative examples of clinical or experimental applications are provided to link conceptual principles to tangible interventions.

### Immune-mediated and ecological tipping points

Immunotherapies provide perhaps the most striking evidence that tumors behave as complex adaptive systems capable of sudden, non-linear transitions. Clinically, some patients exhibit prolonged periods of disease stability followed by abrupt and occasionally complete tumor regression, as documented in cases of spontaneous regression and delayed responses to immunotherapy^59–62^. Such transitions, analogous to phase shifts in ecosystems, highlight the possibility that relatively small immunological perturbations can destabilize the entire tumor collective.

Checkpoint blockade therapies (anti-PD-1, anti-PDL-1, anti-CTLA-4, anti-LAG-3) exemplify this phenomenon. These therapies act by releasing inhibitory signals that constrain antitumor T-cell responses—anti-CTLA-4 enhancing T-cell priming and expansion, and anti-PD-1/PD-L1 restoring effector function in previously suppressed T cells^63,64^. Their effectiveness generally requires pre-existing immune infiltration, as “cold” tumors tend to remain unresponsive. Spatially, cytotoxic lymphocytes infiltrate the tumor mass, fragmenting clusters of immune-evasive or stromal-protected phenotypes and inverting local neighborhood hierarchies^65,66^. The reintroduction of active immune cells shifts cellular dynamics within the tumor microenvironment from a cooperative to an antagonistic state, driven by mechanisms such as cytokine bursts, cytolytic cascades, and metabolic competition. This transformation is not simply additive killing; it represents a topological reorganization of the tumor ecosystem.

From an eco-evolutionary perspective, the immune system operates as a predator guild within a coevolving prey community. Mathematical and computational models of tumor–immune cell dynamics consistently reveal bistability: minor increases in immune predation or antigenicity can flip the system from a stable malignant attractor to extinction^67,68^. Empirical studies also support this framework: immune rejuvenation or ecological perturbations (e.g., infections, fever, or local inflammation) can occasionally trigger spontaneous tumor regression, as documented in historical and clinical observations as well as mechanistic studies of infection-induced immune activation (e.g., refs. ^69–72^).

Importantly, immune-mediated collapse is not only the cumulative result of cell killing, but also a loss of organizational coherence and a shift from a cooperative tumor configuration to a destructive one. Once feedback loops between immune and tumor cells become globally antagonistic, the collective architecture of the tumor disintegrates. This interpretation aligns with ecological theory, where communities exposed to external predation or competition can abruptly transition to depauperate or extinct states once internal feedbacks change sign. Immune activation, in this framework, represents a GPC-based perturbation that transforms the interaction network of the tumor from cooperative to antagonistic, pushing the collective beyond a tipping point toward systemic collapse. Notably, such immune reprogramming is not restricted to immunotherapies: even non-immune treatments such as radiotherapy or certain cytotoxic regimens can break immune tolerance by releasing tumor antigens, inducing inflammatory pathways (e.g., interferon responses), or reducing tumor burden to levels at which tumor-mediated immunosuppression becomes unsustainable^73,74^. These mechanisms further illustrate how diverse therapeutic perturbations can shift the system across critical GPC thresholds.

### Adaptive therapy: sculpting the intra-tumoral ecosystem

Adaptive therapy, pioneered by Gatenby and colleagues^9,75^, is explicitly inspired by ecological and evolutionary principles. Instead of maintaining repeated high-dose treatments, drug administration is dynamically modulated to preserve a subpopulation of sensitive tumor cells that suppress resistant clones through competitive inhibition. This approach was tested clinically in a pilot study of metastatic castrate-resistant prostate cancer, where abiraterone dosing was adapted according to mathematical modeling. Patients managed with this adaptive protocol experienced more than a twofold increase in time to progression, despite receiving substantially lower cumulative drug exposure compared with standard continuous therapy^76^. From a GPC perspective, this success emerges because therapy re-shapes the composition of the tumor community, maintaining a balanced coexistence that prevents the formation of a resistant monoculture. The sensitive-to-resistant ratio, spatial distribution, and resource access collectively define a metastable GPC that becomes unstable once therapy perturbs its balance. When dosing is reduced or paused, the system oscillates around this unstable configuration, analogous to predator–prey dynamics that prevent ecosystem collapse. This re-interpretation suggests a fluctuating regime where no single clone can sustain the malignant collective. In this sense, adaptive therapy can be viewed as a GPC-based strategy that deliberately manipulates the tumor’s internal composition to make the malignant collective resistant to invasion by therapy-resistant clones.

### Differentiation and phenotypic reversion therapies

The paradigm of differentiation therapy originated with all-trans retinoic acid (ATRA) treatment in acute promyelocytic leukemia (APL)^77,78^. In this case, leukemic blasts are not eliminated but induced to differentiate into mature myeloid cells, leading to complete remission. Clinically, this phenotypic reprogramming is associated with profound changes in disease composition, including a rapid decline in immature blasts, restoration of differentiation markers, and dramatic improvements in patient outcomes, with long-term survival rates now exceeding 80–90%^79–81^. Conceptually, ATRA therapy operates on the malignant GPC through a dual mechanism. On one side, it re-injects normal phenotypes that destabilize the functional modules sustaining leukemia and weaken its collective organization. On the other side, it induces massive cell death, an effect shared with immune checkpoint inhibitors, further reducing the tumor’s capacity to maintain a functionally advantageous GPC. As differentiated cells exit the proliferative and cooperative network, the tumor’s group-level organization collapses, rendering its collective function unsustainable. From a GPC perspective, these clinical and biological changes can be interpreted as a longitudinal reconfiguration, and eventual collapse, of the tumor’s phenotypic composition, rather than as the sole consequence of cytotoxic elimination. Leukemias illustrate this principle vividly: when the self-renewal probability of malignant progenitors falls below ~0.5, the population collapses because divisions produce more differentiated than self-renewing cells. No cell killing is required, shifting these odds alone is enough to drive systemic failure of the malignant GPC^82,83^. Although this logic is most intuitive in solid tumors, where spatial architecture plays a central role, emerging evidence also suggests that leukemias display forms of functional cooperation among leukemic stem and progenitor subpopulations. In this sense, differentiation therapy in APL can be seen as perturbing a functionally organized, rather than spatially structured, GPC.

Conversely, BRCA mutant reversion under PARP inhibitor therapy provides a clear example of GPC reconfiguration associated with therapeutic failure: reversion mutations that restore homologous recombination (HR) repair are detected in ~50–80% of BRCA-mutant patients who respond but subsequently relapse (see ref. ^84^ for example). Importantly, BRCA-reversion mutations do not simply reinstate HR; they reorganize the tumor’s phenotypic landscape. PARP-inhibitor–sensitive BRCA-deficient cells typically exhibit a highly proliferative, poorly differentiated, and genomically unstable phenotype. When reversion mutations restore HR, these cells acquire a more stable genome, a distinct metabolic state, and a more differentiated phenotype. This abrupt transition reshapes the internal balance of phenotypes within the tumor, modifying its GPC. As a result, cellular strategies previously suppressed in the BRCA-deficient background (e.g., enhanced survival programs, altered migratory behaviors, or lineage-specific traits) can re-emerge. Therapeutic pressure therefore generates a new GPC in which HR-restored clones gain a selective advantage and ultimately outcompete the original BRCA-deficient population, driving relapse. This example illustrates that not all GPC perturbations lead to collapse; some instead drive the emergence of alternative, therapy-resistant configurations capable of restoring functional coherence.

This principle has since inspired efforts to reprogram solid-tumor cells toward more differentiated or normalized states, using epigenetic modulators, inhibition of mechanotransduction pathways such as YAP/TAZ, or microenvironmental cues promoting partial phenotypic reversion^85,86^. These strategies aim not to eradicate cancer cells directly, but to restore functional order and stability within the tumor ecosystem, thereby reducing malignancy and promoting long-term control.

In GPC terms, these therapies destabilize the phenotype distribution by increasing heterogeneity through differentiation and connectivity. Rather than “curing” through re-education of individual cells, they succeed by fragmenting the cooperative topology of the malignant collective. Differentiation-based therapies thus act as a GPC-based intervention that disrupts the hierarchical organization of the tumor, driving the collective from a structured, self-sustaining configuration toward a functionally neutral or exhausted one.

### Vascular and microenvironmental normalization: reconfiguring the tumor ecosystem

The concept of vascular normalization suggests that instead of “shutting down” blood vessels, certain interventions can transiently restructure the chaotic tumor vasculature into a more functional and perfused network. This results in reduced interstitial pressure, improved oxygenation, and enhanced drug delivery, a phenomenon often termed the *normalization window*^28,87,88^. Anti-VEGF or VEGFR2-targeting agents such as bevacizumab can reduce hypoxia and re-equilibrate nutrient gradients, thereby improving tumor perfusion and sensitizing tumors to radiotherapy and other therapies, as demonstrated in experimental studies of vascular normalization (e.g., refs. ^28,37,89,90^). Although this window is transient, it can be prolonged through rational combinations (e.g., anti-VEGF with TGF-β inhibition), thereby extending the normalized vascular state and enhancing the efficacy of radio- and immunotherapies. However, the limited clinical benefit of anti-VEGF therapies in many contexts highlights that not all spatial GPC perturbations are sufficient to induce durable destabilization, particularly when tumors retain the capacity to re-establish functional gradients and cooperative niches, suggesting that although these therapies alter spatial organization, they often fail to irreversibly disrupt the functional interactions required for tumor persistence.

From a GPC perspective, vascular normalization represents a spatial resource reconfiguration of the malignant collective. By flattening oxygen gradients and redistributing nutrients, it erases ecological niches that shelter resistant phenotypes. The spatial heterogeneity of proliferation, metabolism, and stress responses collapses, pushing the system toward functional homogeneity incompatible with malignant persistence. Moreover, vasculature “normalizing” doses have been shown to reprogram tumor-associated macrophages and enhance immune checkpoint blockade efficacy, linking vascular normalization with a broader immunological normalization of the tumor microenvironment^91,92^.

Beyond blood vessels, the extracellular matrix (ECM) and cancer-associated fibroblasts (CAFs) constitute the structural scaffold through which forces, signals, and metabolites circulate. Remodeling the ECM (e.g., reducing stiffness or crosslinking) or targeting focal adhesion kinase (FAK) can lower interstitial pressure, improve macromolecule penetration, and disrupt cooperative modules within the tumor, thereby destabilizing the GPC. Recent findings show that FAK inhibition can homogenize the tumor microenvironment and enhance antibody–drug conjugate uptake^93^, while indiscriminate CAF depletion can paradoxically worsen disease (e.g., in pancreatic ductal adenocarcinoma) by increasing immunosuppression^94^. Likewise, proteases such as matrix metalloproteinases (MMPs) and cathepsins, by reshaping ECM composition and organization, can profoundly influence interstitial pressure, diffusion, and cell–cell communication^95^. Together, these observations highlight the importance of modulatory rather than destructive strategies, *ecological tuning* instead of *environmental eradication*.

In summary, vascular and microenvironmental normalization do not act primarily by killing cells but by reconfiguring the tumor architecture that sustains cooperation among them. In GPC terms, these interventions disrupt functional connectivity and reduce spatial diversity, driving the malignant collective toward systemic instability, a pathway to collapse that amplifies the effects of other therapies. Vascular and stromal normalization therefore function as GPC-disrupting approaches that rewire the spatial and resource architecture of the tumor, erasing ecological niches that sustain cooperative resistance and collective survival.

Within the broader GPC framework, immune activation, together with adaptive dosing, differentiation therapy, and vascular normalization, can all be understood as distinct but convergent routes to GPC destabilization. These different anti-cancer strategies target distinct axes of the tumor’s functional architecture (composition, spatial organization, or interaction network), each promoting the same emergent outcome: the collapse of collective stability. However, in metastatic disease these perturbations rarely eradicate all malignant organization. Even when therapies transiently destabilize the prevailing GPC, residual subpopulations can persist, through dormancy-like states, immune evasion, metabolic resilience, or therapeutic tolerance, and subsequently reorganize into a new, functionally coherent GPC. This capacity to rebuild collective functionality after partial collapse provides a mechanistic basis for relapse in most advanced cancers and is consistent with recent theoretical work proposing that tumors can transition between alternative GPC states through cycles of destabilization and reassembly^96^.

### Extinction therapy and multi-strike strategies

Although our perspective focuses on *durable control* rather than complete eradication, the GPC framework can also be naturally extended to therapeutic approaches explicitly designed to eliminate tumors. Gatenby and colleagues have recently proposed the so-called *extinction therapy* or *multi-strike strategy*^23,97^, which aims to exploit the eco-evolutionary vulnerability of small and/or declining cancer populations. In this view, eradication becomes possible when successive therapeutic “strikes” push the tumor below critical thresholds of population size (Allee thresholds) or functional organization, making recovery impossible. From a GPC standpoint, extinction therapy can be interpreted as deliberately driving the malignant collective across a terminal tipping point: repeated perturbations progressively erode compositional diversity, weaken cooperative functional modules, and dismantle spatial organization until the tumor is no longer able to maintain a viable group-level phenotype.

A recent mathematical analysis of this strategy^98^ further supports this logic by showing that multi-strike regimens are most effective when they exploit intrinsic lags in the tumor’s ability to rebuild cooperative interactions after perturbation. The probability of extinction increases sharply when treatment strikes are optimally timed to prevent the tumor re-establishing key functional modules, for example, metabolic complementarity, resistance-sharing behaviors, or niche-engineering structures that maintain a favorable microenvironment.

From a GPC perspective, this is a critical point: what these models implicitly capture is not merely a reduction in cell number, but the transient fragility of the tumor’s group-level phenotype. After each strike, the malignant collective must “reorganize”, reconstructing spatial coherence, reconstituting cooperative networks, and restoring phenotypic heterogeneity that supports division of labor. If the next perturbation occurs before this reassembly is complete, the tumor is forced into an increasingly disorganized state, eventually falling below the viability boundary required to sustain collective functionality.

## Quantifying GPC transitions and critical points

### Detecting, characterizing and monitoring GPC transitions

If the destabilization of the GPC truly underlies durable therapeutic success, then the next conceptual and practical challenge is to detect and characterize these transitions. Advances in single-cell and spatial omics, combined with multiplexed imaging and computational network inference, now make it possible to detect and monitor how therapeutic perturbations reshape tumor ecosystems at multiple scales^66,99–101^. Importantly, a growing body of empirical work already demonstrates that key dimensions of the GPC are measurable and clinically informative. For example, single-cell and TCR sequencing studies have identified distinct tumor immune microenvironment states associated with differential responses to chemo-immunotherapy^102^, highlighting the predictive value of compositional GPC features. Similarly, spatial proteomics and imaging mass cytometry have revealed that the spatial organization, activation state, and neighborhood structure of tumor and immune cells can predict therapeutic response and clinical outcome^103–105^. In several cancer types, spatial archetypes of tumor architecture and specific immune–tumor configurations have been directly linked to prognosis and treatment sensitivity^106^. At the functional level, recent studies using single-cell transcriptomics have shown that therapy can alter ligand–receptor interaction networks between tumor and immune cells^107,108^, illustrating the disruption of cooperative interactions that are central to the GPC framework. Together, these findings indicate that GPC dimensions (compositional, spatial, and functional) are not only theoretically defined but already partially quantifiable in clinical contexts. These studies collectively demonstrate that the GPC framework is not introducing entirely new measurable entities, but rather provides an integrative lens to interpret already quantifiable dimensions of tumor organization. In addition to *transcriptomic* and *imaging* techniques, *proteomics*—especially LC-MS/MS-based spatial proteomics—serves as a powerful tool for quantifying the functional outputs of tumor systems. Proteomic profiling allows for the detection of changes in signaling pathways, post-translational modifications, and intercellular communication networks, which can indicate GPC destabilization even when transcriptomic data is unclear. As mentioned before, alterations in the epigenome or the *epitranscriptome* have been identified as significant regulators of cellular plasticity and lineage transitions^109^. Combining these insights with spatial and proteomic data could begin to approximate indicators of critical transitions and the loss of organizational coherence within tumors.

### Quantifying GPC changes

Across these datasets, several quantitative dimensions may serve as preliminary or partial early-warning indicators of a system approaching a tipping point. In the *compositional dimension*, high or rising phenotypic diversity, captured through measures such as Shannon entropy or clonal frequency distributions derived from single-cell RNA sequencing datasets, can reflect the loss of hierarchical organization within the tumor. Rather than signaling a linear increase toward collapse, this elevated diversity marks a state of phenotypic plasticity and instability often observed in perturbed or therapy-responsive tumors^110–112^. However, at present, these measurements remain proxies, and no existing method can capture the full architecture of GPC transitions without integrating prior biological knowledge.

Similarly, within the *spatial dimension*, loss of autocorrelation and local clustering, detected using spatial statistics such as Moran’s *I* or Ripley’s *K* on imaging mass cytometry or CODEX data, reveals the disintegration of the organized tissue architecture that supports malignancy^66,113^. These approaches have already been successfully applied to identify spatially organized cell neighborhoods associated with prognosis and treatment response^104,105^. While spatial analyses such as Moran’s *I* or Ripley’s *K* reveal the disintegration of organized tissue architecture, studies combining microdissection with multi-regional sequencing have shown that genotypic heterogeneity does not necessarily translate into functional diversity. Distinct clones may converge toward similar phenotypic or metabolic states under shared ecological constraints (e.g., refs. ^114,115^). This highlights that GPC destabilization operates at the level of functional organization rather than clonal identity. Beyond transcriptomic and proteomic changes, emerging work also highlights the contribution of epitranscriptomic regulators, such as m⁶A-dependent RNA methylation, which modulate lineage plasticity and therapy-induced state transitions, thereby influencing GPC stability^116,117^. By mapping epitranscriptomic landscapes across different tumor subpopulations, one can uncover hidden regulators of GPC stability and identify predictive indicators of when a tipping point may be reached.

At the *functional level*, cell–cell interaction networks inferred from ligand–receptor co-expression (e.g., using CellPhoneDB, NicheNet, or Squidpy) can exhibit a drop in network density and modularity, reflecting the fragmentation of cooperative modules essential for tumor viability^100,101,118^. Likewise, the *resource dimension*, characterized through hypoxia mapping, vascular density, and perfusion gradients obtained from MRI, PET, or histological approaches, tends to undergo homogenization as spatial niches collapse and metabolic differentiation vanishes^90,119^. Finally, the *temporal dimension* provides dynamic early-warning signals such as rising variance and autocorrelation, phenomena known as *critical slowing down*, indicating that the system is losing resilience and recovering more slowly from perturbations^31,120^.

### Predicting transitions and critical points

Together, these compositional, spatial, functional, resource, and temporal signatures define a quantitative ecology of tumors. Their coordinated tracking can reveal whether a therapy is merely shrinking tumor mass or actually pushing the malignant collective across a functional threshold. In practice, integrating these indicators may allow clinicians to detect when a tumor’s internal organization is collapsing, a shift from cytotoxic monitoring toward a focus on loss of collective coherence as a biomarker of therapeutic success. Ultimately, the goal is to use such information to understand and predict GPC transitions. Achieving this goal will require coordinated advances along three complementary axes: (i) the generation of longitudinal, multi-scale datasets capturing the compositional, spatial, and functional dimensions of tumor organization; (ii) the development of integrative and mechanistic models capable of linking these data to non-linear system dynamics and predicting tipping points; and (iii) the validation of candidate early-warning indicators across experimental and clinical settings. This remains a challenging task, as it requires prior knowledge of network interactions and may involve AI-mediated inference of multiple contributing factors.

## Conclusion

Cancer therapy has long been guided by the principle that killing more cells yields better outcomes. Yet across diverse contexts, durable control frequently arises from interventions that do not eradicate tumors but rather disrupt their collective organization. Reinterpreting such responses through the lens of the GPC reveals a unifying logic: successful therapies, whether adaptive, differentiating, vascular, or immune-based, often act by destabilizing the internal architecture of the malignant consortium (Table 1).

Beyond this unifying interpretation, our framework identifies general principles that distinguish transient from durable therapeutic outcomes. In this view, therapeutic success corresponds to the collapse of functional coherence rather than the annihilation of individual cells. Tumor stability thus depends not only on its genetic composition but on the maintenance of specific spatial, metabolic, and communicative structures that sustain cooperation among its components. Once these configurations are pushed beyond a tipping point, the system loses its group-level stability even when many constituent cells remain alive. By formalizing the concept of GPC, this framework unites evolutionary, ecological, and systems perspectives into a single principle linking tumor organization, stability, and therapeutic response.

Recent experimental work further illustrates this logic. A proof-of-concept study showed that colon cancer cells could be reprogrammed into normal-like phenotypes through the activation of molecular network switches^121^. While such findings were presented as cellular reversal, complete conversion of all malignant cells is unlikely to be necessary. Even partial reprogramming, restoring normal functionality in a subset of tumor cells, would profoundly alter the GPC by reshaping intercellular interactions, feedback loops, and resource flows. From this viewpoint, such approaches are better understood as GPC-based interventions that reconfigure system-level organization rather than revert individual cell identities.

Translating this framework into practice will require defining measurable proxies for GPC stability, validating them across tumor types, and understanding how therapeutic interventions reshape these higher-order dynamics. More broadly, viewing cancer as a dynamic consortium structured by its GPC could illuminate not only therapy response but also the emergence of resistance, dormancy, and metastatic competence.

Recognizing, monitoring, and ultimately inducing such GPC destabilization may therefore represent a new frontier for oncology. Integrating spatial and temporal omics, ecological modeling, and early-warning metrics could make it possible to detect when a tumor approaches critical instability. Designing therapies that target the organization of the system rather than its parts could finally align cancer treatment with the logic of complex adaptive systems—where the key to control lies not in destruction, but in the art of functional destabilization. In doing so, oncology paradigms may evolve from the science/goal of eradication to the science/goal of systemic reorganization.

## Data Availability

No datasets were generated or analyzed during the current study.
